# Causal Inference for Hypertension Prediction With Wearable E lectrocardiogram and P hotoplethysmogram Signals: Feasibility Study

**DOI:** 10.2196/60238

**Published:** 2025-01-23

**Authors:** Ke Gon g, Yifan Chen, Xinyue Song, Zhizhong Fu, Xiaorong Ding

**Affiliations:** 1 School of Life Science and Technology, University of Electronic Science and Technology of China, Research Building C348A, 3rd Fl, Chengdu, 611731, China, 86 18030493605

**Keywords:** hypertension, causal inference, wearable physiological signals, electrocardiogram, photoplethysmogram

## Abstract

**Background:**

Hypertension is a leading cause of cardiovascular disease and premature death worldwide, and it puts a heavy burden on the healthcare system. Therefore, it is very important to detect and evaluate hypertension and related cardiovascular events to enable early prevention, detection, and management. Hypertension can be detected in a timely manner with cardiac signals, such as through an electrocardiogram (ECG) and photoplethysmogram (PPG) , which can be observed via wearable sensors. Most previous studies predicted hypertension from ECG and PPG signals with extracted features that are correlated with hypertension. However, correlation is sometimes unreliable and may be affected by confounding factors .

**Objective:**

The aim of this study was to investigate the feasibility of predicting the risk of hypertension by exploring features that are causally related to hypertension via causal inference methods. Additionally, we paid special attention to and verified the reliability and effectiveness of causality compared to correlation.

**Methods:**

We used a large public dataset from the Aurora Project , which was conducted by Microsoft Research. The dataset included diverse individuals who were balanced in terms of gender, age, and the condition of hypertension, with their ECG and PPG signals simultaneously acquired with wrist -worn wearable devices. We first extracted 205 features from the ECG and PPG signals, calculated 6 statistical metrics for these 205 features, and selected some valuable features out of the 205 features under each statistical metric. Then, 6 causal graphs of the selected features for each kind of statistical metric and hypertension were constructed with the equivalent greedy search algorithm. We further fused the 6 causal graphs into 1 causal graph and identified features that were causally related to hypertension from the causal graph . Finally, we used these features to detect hypertension via machine learning algorithms.

**Results:**

We validated the proposed method on 405 subjects. We identified 24 causal features that were associated with hypertension. The causal features could detect hypertension with an accuracy of 89%, precision of 92 % , and recall of 82%, which outperformed detection with correlation features (accuracy of 85%, precision of 88 % , and recall of 77%).

**Conclusions:**

The results indicated that the causal inference –based approach can potentially clarify the mechanism of hypertension detection with noninvasive signals and effectively detect hypertension. It also reveal ed that causality can be more reliable and effective than correlation for hypertension detection and other application scenarios.

## Introduction

Hypertension, also known as high blood pressure (BP), is a condition in which the pressure of the blood increases in the arteries. The diagnosis of hypertension relies on BP measurement, and it is defined as systolic BP (SBP) ≥ 140 mmHg or diastolic BP (DBP) ≥ 90 mmHg [1]. Hypertension can be further classified into 3 stages. Stage 1 hypertension is associated with SBP and DBP ranges of 140 ‐ 159 mmHg and 90 ‐ 99 mmHg, respectively. Stage 2 hypertension is characterized by SBP and DBP ranges of 160 ‐ 179 mmHg and 100 ‐ 109 mmHg, respectively. For stage 3 hypertension, the SBP and DBP are more than 180 mmHg and 110 mmHg [1,2].

Furthermore, it is noteworthy that even when SBP ≥ 115 mmHg and DBP ≥ 75 mmHg, a continuous relationship exists between the increase in BP level and the occurrence of cardiovascular or renal pathological conditions and even fatal events. The definition of high blood pressure as SBP ≥ 140 mmHg or DBP ≥ 90 mmHg primarily serves the purpose of simplifying hypertension diagnosis and decision -making regarding hypertension treatment. This threshold was chosen because the benefits of intervention outweigh the risks associated with nonintervention in this context.

According to a review of the global epidemiology of hypertension [3], hypertension is a leading preventable risk factor for cardiovascular disease and all-cause mortality worldwide. In 2010, a total of 1.38 billion people (31.1 % of the global adult population) had hypertension. The prevalence of hypertension is rising globally owing to the aging of the population and increases in exposure to lifestyle risk factors, including unhealthy diets and lack of physical activity.

In addition, hypertension can be divided into primary and secondary forms. Secondary hypertension originates from specific causes and only encompasses a small fraction of the population. Primary hypertension covers the remaining large fraction of the hypertension population, and it arises from intricate interactions among genetic factors, environmental influences, and the aging process. These factors collectively contribute to an increase in systemic vascular resistance, a hallmark hemodynamic abnormality that leads to elevated BP in almost all hypertensive individuals [4]. Furthermore, considering that hypertension may not show any symptoms in its early stages and that there is a continuous relationship between an increase in BP and the risk of stroke, coronary heart disease ,heart failure , and chronic kidney disease , it is very important to detect and treat hypertension in the early stages.

Moreover, physicians often diagnose hypertension by office BP, but masked hypertension and white coat hypertension cannot be effectively detected by office BP. Instead, they usually detect masked hypertension and white coat hypertension through a 24-hour ambulatory recording of the BP signal [5] , but this process is cumbersome. Hence, there are data-driven approaches based on noninvasive signals for the detection of hypertension, such as electrocardiogram (ECG) or photoplethysmogram (PPG), that are easily accessible from wearable sensors [2]. Subsequently, wearable monitoring can continuously monitor patients ’ physiological conditions 24 hours a day. Compared with outpatient blood pressure monitoring, wearable monitoring can obtain patients ’ rhythm information and real physiological conditions (to avoid white coat hypertension and other conditions), as well as the impact of patients ’ behaviors on physiological indicators and other personalized information. Rich reference information is conducive to more accurate assessment and stratification of individual risks.

There are various studies on detecting hypertension with data -driven methods based on noninvasive signals. These methods include classic machine learning models with hand -extracted features and feature representation learning with deep learning methods. For example, Paragliola et al [6] proposed a novel approach for analyzing and classifying the ECG signal with a hybrid deep learning network method called hybrid deep network , which combines long short- term memory ,convolutional neural networks , and deep neural networks . The hybrid method can reach an average accuracy of 0.98 and an average sensitivity and specificity of 0.97. Elgendi et al [7] reviewed the effect of different types of artifacts added to the PPG signal, characteristic features of the PPG waveform, and existing indexes on hypertension diagnosis. In another study, Alkhodari et al [8] used features related to heart rate variability to predict hypertension based on decision trees and random undersampling boosting. The accuracy of the method was 0.81, with the *F*_1_ -score and area under the receiver operating characteristic curve (AUC) being 0.86 and 0.89, respectively. In a study about the automated detection of hypertension severity, Rajput et al [9] developed a 2-band optimal orthogonal wavelet filter bank method, which generates 6 subbands from each ECG signal through a 5-level wavelet decomposition. Further, the sample mean and wavelet entropy features of all subbands were computed to predict the risk of hypertension with classic machine learning methods, such as k-nearest neighbors and support vector machine , and the proposed method can achieve an average classification accuracy of 0.99.

However, most of the previously mentioned studies rel ied on extracting features correlated with hypertension but ignored the causality of hypertension and characteristic variables. Due to the presence of confounding factors, correlations can lead to wrong conclusions, just like Simpson’s paradox [10]. In different populations, the distribution of confounding factors will change, which means the correlations can be unstable and unreliable. Instead, causal inference can not only identify more reliable feature variables with the elimination of confounding factors but also provide more trustworthy guidance for further exploring the physiological mechanisms of hypertension [11].

**Figure 1.**
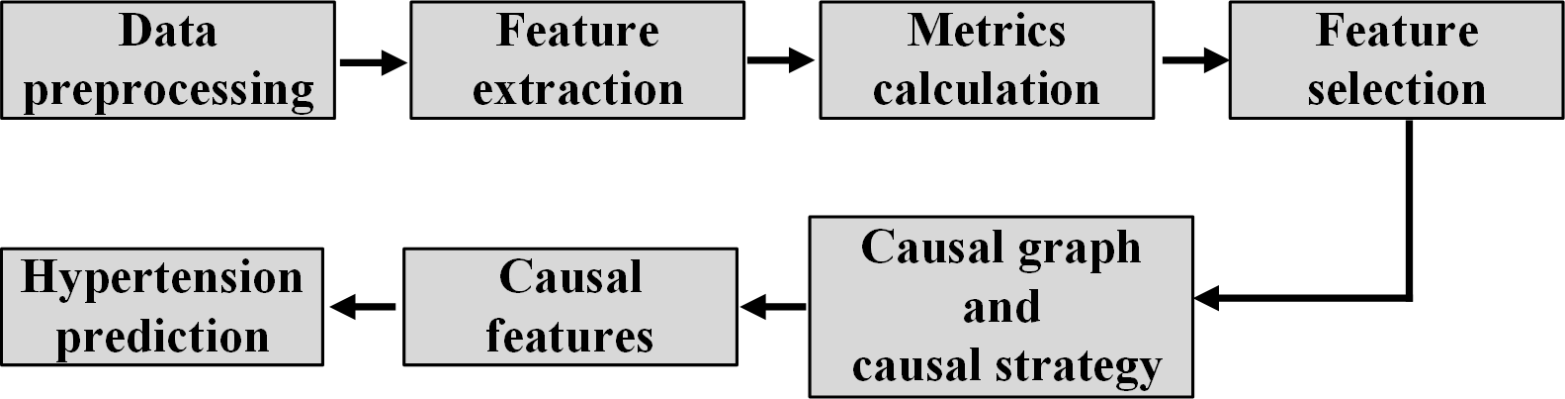
Research route flow char t.

In this work, we propose to predict hypertension based on causal inference with wearable noninvasive signals. The overview of the proposed method is delineated in Figures1  2. We will select effective features based on causality between hypertension and features extracted from PPG and ECG signals. Then, combined with the detected causal features, we will predict hypertension and evaluate its prediction performance by various evaluation metrics. Ultimately, we aim to identify some features that may be of great value in predicting hypertension.

**Figure 2 .**
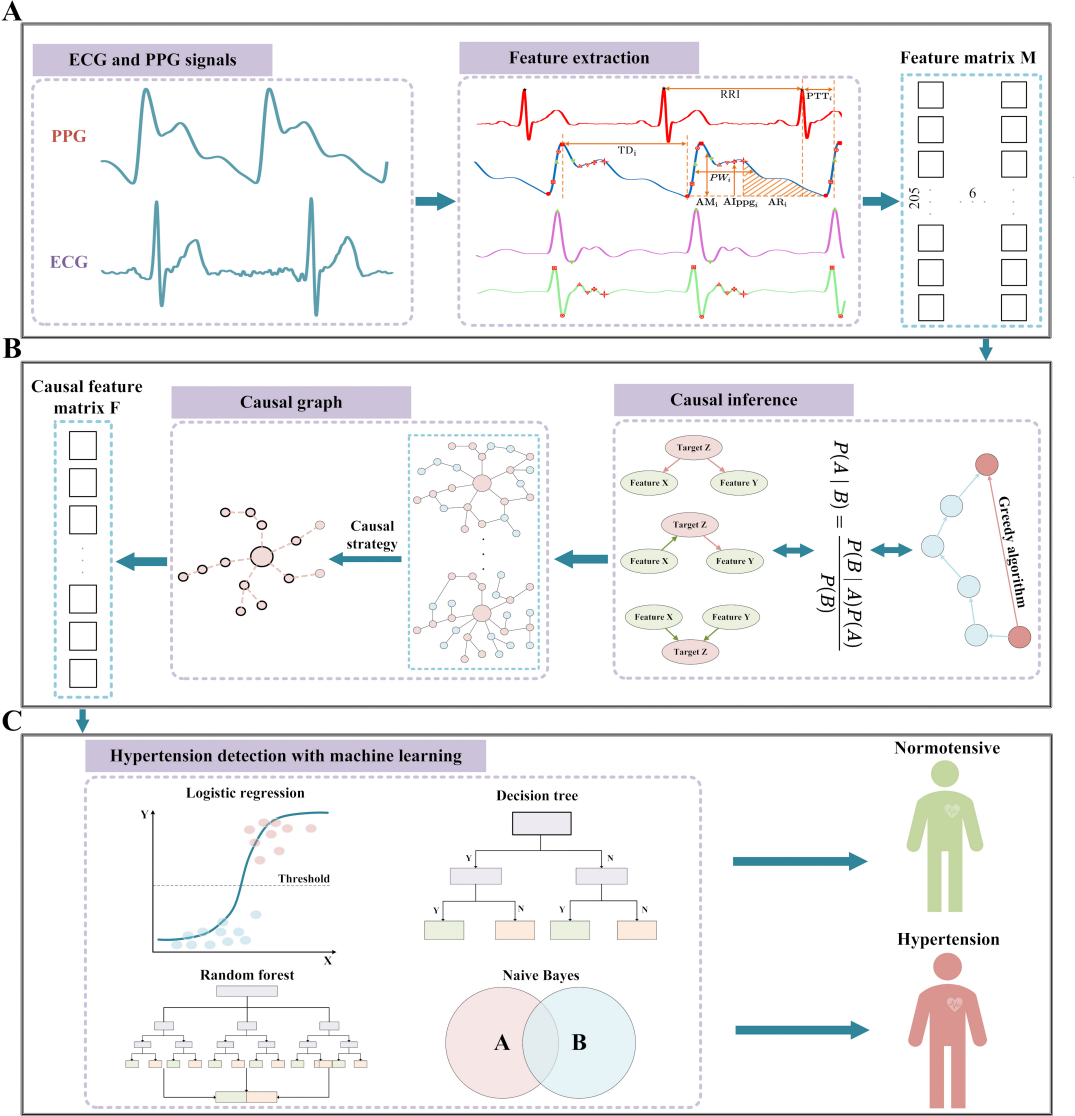
Flowchart of the causal inference for hypertension prediction. (A) Signal preprocessing: 205-dimension beat-by-beat features were extracted from the ECG and PPG as well as the first and second derivatives of the PPG signal (dPPG, sdPPG), and the statistical metrics of these features were calcu lated as the feature matrix M . (B) Based on the feature matrix M, the causal graphs of the extracted features and hypertension status were identified with the causal inference algorithm (the equivalent greedy search algorithm) . (C) The causal feature matrix F was identified from the causal graph obtained from step (B), and we used machine learning classification algorithms to achieve hypertension prediction. ECG: electrocardiogram; PPG: photoplethysmogram.

## Method s

The methods of this paper can be divided into 7 steps ; the details of each step are shown in Figure 1.

### Ethical Considerations

In this study, we used data from the Microsoft Waveform Database, and we obtained data access permission from the Microsoft Data Access Committee [12].Microsoft obtained institutional review board approval from WCG IRB (Puyallup, WA, United States). Individuals unable to consent in English, pregnant women, prisoners, institutionalized individuals, and individuals younger than 18 years were excluded from participation due to their vulnerable status. All the subjects voluntarily participated in the experiment and signed informed consent. The original informed consent and the institutional review board both allow for secondary analysis without additional consent. The dataset used in this study was de- identified to protect the privacy of the subjects.

### Data

The database that we obtained data from was developed for validating new methods for blood pressure measurement with noninvasive sensors. Noninvasive epidermal pressure signals, ECG signals , and PPG signals were acquired with tension, electrical, and optical sensors, respectively. Meanwhile, the reference blood pressure was measured with either the oscillometric method or the auscultatory method. In this study, we used noninvasive signals for hypertension detection. To validate our proposed method, we used data collected based on the oscillometric method. A total of 614 subjects participated in the oscillometric protocol scheme, with ages ranging from 18 -85 years. After excluding data anomalies during the collection process, including miswear, malfunction, data file failure, participant opt-out, alignment failure, and quality failure , relevant measurement information from 483 subjects was retained [12]. In a further waveform preprocessing step, poor waveform segments and subjects with less than 4 qualified waveform segments were removed , which led to the final retention of measurement data from 405 participants, comprising 183 hypertensive patients and 222 healthy individuals. The ages of the 405 participants ranged from 18 -60 years, with an average age of 45 years . In addition, the 405 participants comprised 199 females and 206 males.

Moreover, measurements in this protocol were obtained during controlled laboratory visits spaced at least 24 hours apart. Additionally, dynamic measurements were collected during the 24-hour interval between laboratory visits. Automatic measurements were taken every 30 minutes in the morning and every 60 minutes in the evening. Each patient typically had 24 -36 waveform segments, with each acquired for 15 -30 seconds. Our feature extraction primarily relied on data from dynamic measurements.

### Feature Extraction

**Figure 3.**
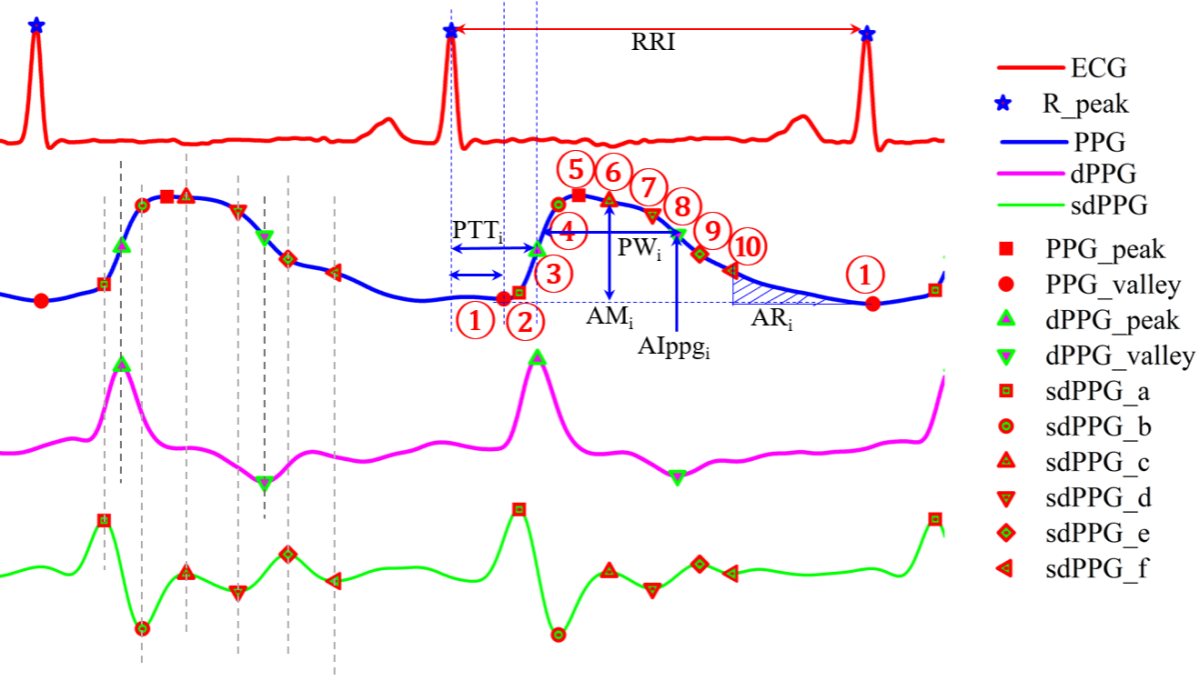
Diagram of fiducial points of the ECG and PPG signals as well as major types of features [13] . AI: absolute intensity; AR: area under the PPG curve; AM: amplitude; dPPG: the first derivative of PPG; ECG: electrocardiogram; PPG: photoplethysmogram; PTT: pulse transit time; PW: pulse width; RRI: R-R interval; sdPPG: the second derivative of PPG .

We extracted 205 features from the filtered ECG and PPG signals with the extraction method defined in our previous study [13]. The features mainly include pulse transit time (PTT),time duration (TD), amplitude (AM), intensity of PPG, the first derivative of PPG (dPPG), the second derivative of PPG (sdPPG), area under the PPG curve (AR) , and physiological meaningful relative index (RI). The mathematical expression and definition of the se features are as follows and are also described in Table 1 . The fiducial points of ECG, PPG, dPPG, and sdPPG signals of each cardiac cycle were identified to calculate the features. The identified fiducial points are illustrated in Figure 3.

**Table 1.** Features extracted from electrocardiogram and photoplethysmogram signal s.

Index	Classification	Definition of features
1 ‐ 10	Pulse transit time	Time deviation between R peak of electrocardiogram and fiducial points of photoplethysmogram
11 ‐ 66	Time duration	Time duration between 2 fiducial points of photoplethysmogram
67 ‐ 111	Amplitude	Amplitude between fiducial points of photoplethysmogram
112 ‐ 130	Pulse intensity	Intensity of photoplethysmogram, dPPG^a^,and sdPPG^b^ at fiducial points
131 ‐ 185	Area	Area under the photoplethysmogram curve between fiducial points
186 ‐ 205	Relative index	Physiological meaningful ratio index

a dPPG: the first derivative of photoplethysmogram.

b sdPPG: the second derivative of photoplethysmogram.

*Feature Point (FP, 1 *∼* 10 *) = [PPG valley, sdPPG a, dPPG peak, sdPPG a, PPG peak, sdPPG c, sdPPG d, dPPG valley, sdPPG e, sdPPG f, PPG valley next]

*PTT* =FP(i) - R peak, i = 1∼10

*TD* = [RRI, (FP(j) - FP(i)), i,j = 1∼10, and *j >* *i* ]

*AM* =PPG(FP(j)) - PPG(FP(i)), i = 1∼10, and *j >* *i*

*AIPPG* =PPG(FP(i)), i = 1∼10

*AIdPPG* =dPPG(FP(i)), i = 1∼10

*AIsdPPG* =sdPPG(FP(i)), i = 2,4,7∼10

*AR* =Area between (FP(j) – FP(i)), i,j = 1∼10

*RI*: relative rising time, dicrotic diastolic ratio, augmentation index, inflection point area point, slope transit time, ratio of sdPPG (b/a, c/a, (c + d –b)/a, etc), PPG intensity ratio, perfusion index [13].

After obtaining the above features, we can perform feature selection and build a causal graph based on the causal inference algorithm.

### Algorithm of Causal Inference

**Figure 4.**
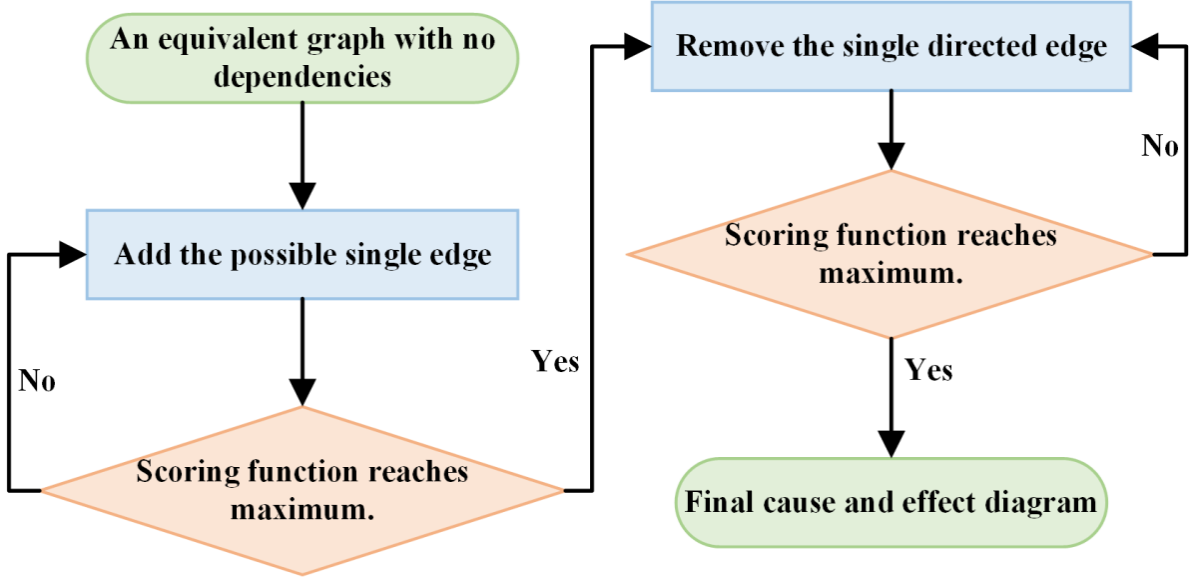
Flowchart of the greedy equivalence search algorithm.

We used the greedy equivalence search (GES) algorithm to learn the causal graph. The GES algorithm is based on the theoretical basis of Meek’s conjecture [14]. The Meek’s conjecture is: if direct acyclic graph (DAG) M is an independent map of another DAG F, then there exists a finite set of edges in DAG F that can be added or reversed , after each modifiable edge is added or reversed direction, DAG M is still an independent graph of DAG F. After all modifications are done, M = F. Underlying the Meek’s conjecture, we can use generalized score functions [15] and the GES algorithm to get the final causal graph. Figure 4 shows the implementation steps of the GES algorithm. In addition, we also provide the pseudo code to illustrate the detailed steps of the GES algorithm as shown in Textbox 1 .

Textbox 1. **Algorithm 1: Apply-edge-operation(G ,H ).
**

Input: $DAG_{s}$ G and H where G≤H and G≠H

1: Set $G^{\prime }\leftarrow G$

2: While G and H contain a node Y that is a sink node in both $DAG_{s}$ and for which $Pa_{Y}^{G}=Pa_{Y}^{H}$ , remove Y and all incident edges from both $DAG_{s}$

3: end while

4: Let Y be any sink node in H

5: if Y has no children in G then

 6: Let X be any parent of Y in H that is not a parent of

 7: Y in G , add the edge X→Y

 8: return $G^{\prime }$

9: end if

10: Let $De_{Y}^{G}$ denote the descendants of Y in G

11: And let D ∈ DeY G denote the (unique) maximal element from this set within ℋ2

12: Let Z be any maximal child of Y in G such that G is a descendant of Y in G

13: if Y→Z is covered in G

 14: reverse Y→Z in $G^{\prime }$

 15: Return $G^{\prime }$

16: end if

17: if There exists a node X that is a parent of Y but not a parent of Z in $G^{\prime }$ then

 18: add X→Z to $G^{\prime }$

 19: return $G^{\prime }$

20: end if

21: Let X be any parent of Z that is not a parent of Y

22: Add Y→Y to $G^{\prime }$

23: return $G^{\prime }$

Output: DAG $G^{\prime }$ that results from adding or reversing an edge in G .

Then, the GES algorithm has 2 stages. In the first stage, it starts from an equivalence class (empty graph) with no dependencies and keeps adding possible edges to search for the largest equivalence class of generalized scoring functions until the scoring functions’ local maximum is reached. Then, in the second stage, the greedy principle is used to gradually delete the directed edges until the generalized scoring function reaches the local maximum again, and the final causal graph is obtained.

Considering that hypertension is a discrete variable while the feature variables are continuous, we are essentially dealing with mixed data. Traditional scoring functions such as Bayesian information criterion and Bayesian Dirichlet equivalent uniform do not take into account the issue of mixed data; for example, it discretizes continuous data and process it uniformly, resulting in a loss of valuable information. Therefore, we introduce a generalized scoring function to replace traditional scoring functions. The generalized function is primarily based on kernels and handles linear causal relationships, nonlinear causal relationships, continuous variables, discrete variables, and mixed data in a uniform manner, maximizing information retention. Finally, this scoring function addresses the issue of Markov equivalence classes, to some extent, overcoming the limitation of equivalence greedy search algorithms in distinguishing Markov equivalence classes.

Finally, we needed to organize a feature matrix in which each row represents a sample and each column represents a kind of feature, then input this matrix into the equivalent greedy search algorithm to obtain the causal graph. Prior to this, feature selection is a necessary step to construct the feature matrix.

### Feature Selection

This section mainly explains the specific process of feature selection in this study, which is mainly divided into the following 3 parts. After completing feature selection, we will perform causal strategy and causal graph construction.

**Figure 5.**
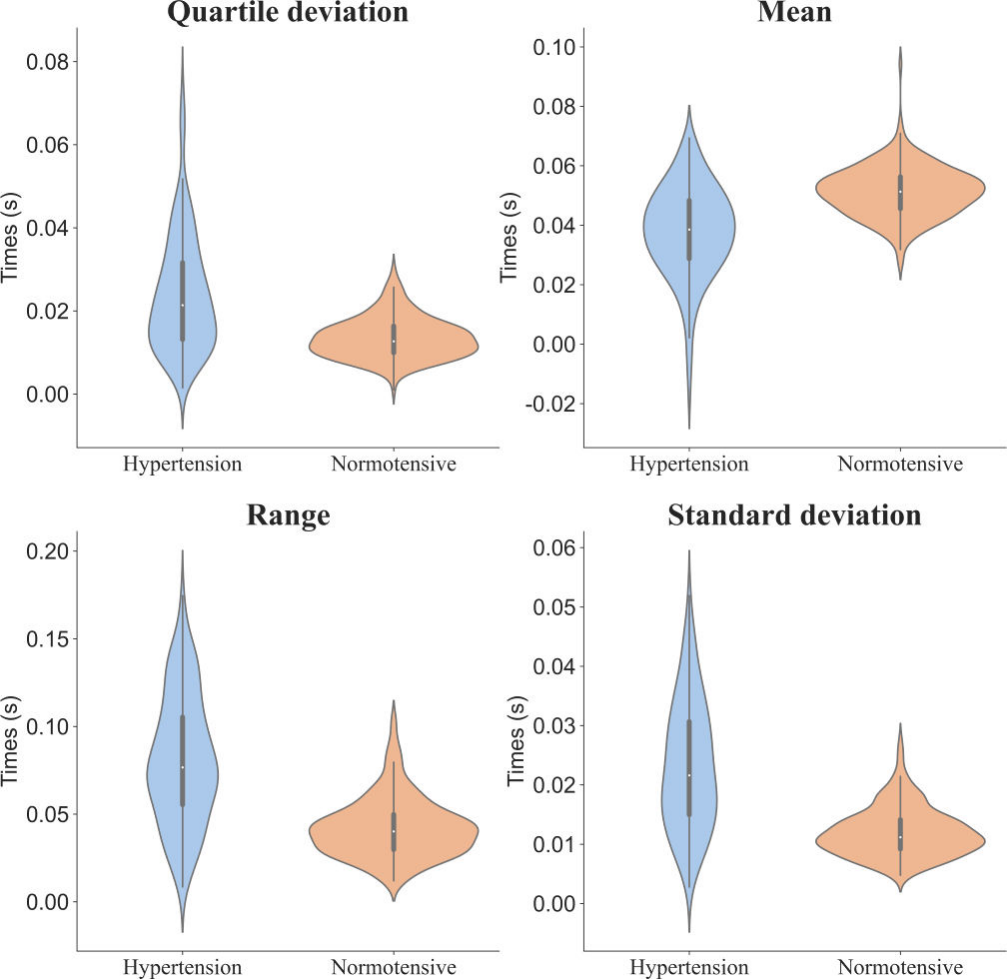
Box plot of the various statistical indicators of the feature 52.

1. Six statistical metrics: Since ECG and PPG signals a re time series data, we extracted the beat-by-beat features and calculated the statistical metrics of these 205 features to represent the temporal variability information. The statistical metrics include: standard deviation , range, mean, quartile deviation , coefficient of variation, and median, which result in 205 ×6 = 1 230 dimensional features. This allows us to capture and analyze the temporal characteristics of ECG and PPG signals while summarizing them using key statistical measures. Based on the extracted features, we then detected the 6 different causal graphs of these features with hypertension, which provide insights into the relationships and causal effects among the extracted feature variables and hypertension.
2. Significant difference analysis: Now, we need to use the corresponding 205 features to construct a causal graph under each metric. Due to the limitations of the equivalent greedy search algorithm calculation efficiency, hardware device computing power resources, and the number of subject samples, the time cost of constructing a causal graph based on 205 features is unacceptable. Therefore, we will use significant difference analysis to exclude features that do not show significant differences between hypertensive patients and healthy people. Then, considering the time cost and sample size, we will sort the retained features according to the degree of significant difference. We ultimately selected less than 50 features for causal graph construction.
3. Causal feature selection: In the following, we select the features that have a direct causal relationship with the hypertension node from the causal graph constructed under each metric. A total of 24 causal features were selected under the 6 metrics. It should be noted here that different metrics mean observing the changes of the same feature over a period of time from different perspectives. The features with the same number under different statistical metrics are essentially derivatives of the original features. Taking feature 52 as an example, we can get 4 feature variables under these metrics ; they are shown in Figure 5 . These 4 feature variables are essentially derivatives of feature 52. Therefore, in the final causal graph , we use feature 52 nodes to represent the above 4 features. From this, we can see that there are some features with the same number among the 24 causal features. We can use a feature node in the final causal graph to represent these feature variables with the same number, and finally obtain a final causal graph containing 10 feature nodes.

### Strategy of Causal Inference

In order to mitigate the potential issues of bidirectional causality and cyclic graphs, we conducted the analysis of the causal relationships between respective feature variables and hypertension under each indicator, culminating in the derivation of corresponding causal subgraphs, so as to obtain the causal graph.

1. Strategy for obtaining causal graph: We randomly partitioned the dataset to identify the causal graph, with the allocation of an additional validation set for subsequent hypertension risk prediction. Recognizing that a single random partitioning could introduce undesired stochasticity (thereby rendering the resulting causal graphs potentially unrepresentative), we draw inspiration from the concept of 10-fold cross-validation. This method involves conducting 10 iterations to compute causal subgraphs, followed by a rigorous pruning process to retain only those segments demonstrating direct causal associations with hypertension within each causal subgraph. Subsequently, guided by the principle of majority rule, we amalgamate the results of these iterations to derive the ultimate causal subgraph.
2. Strategy for merging causal graph: After obtaining the final causal subgraph with each graph identified with the 6 categories of features mentioned in feature selection section , we assume that the weights of the causal relationships between the feature variables and hypertension are equal under each category of feature ; based on the principle of majority rule, we integrate multiple causal subgraphs into the ultimate causal graph. This method can screen out more reliable direct causal feature variables, further simplify the causal graph, and preserve important information.

### Classifier and Performance Evaluation

In conjunction with a 10-fold cross-validation approach to partition the dataset into training and testing sets, our predictive modeling of hypertension risk primarily leverages 4 classification algorithms: random forest, logistic regression, decision trees, and naive Bayes. These algorithms are selected for their effectiveness in capturing diverse patterns in the data. Moreover, the evaluation of our models is based on a comprehensive set of performance metrics, encompassing accuracy, precision, recall, *F*_1_ -score, and the AUC , which are defined later on. Following the derivation of the final causal diagram, we proceeded to select an equal number of feature variables with the strongest correlation to hypertension, based on the point-biserial correlation coefficient. These selected features were then used in the prediction of hypertension risk. Subsequently, we compared the predictive performance of this model with the one based on causal feature variables.

## Results

### Signal and Feature Analysis

We found that there are 24 feature variables directly causally related to hypertension under 6 indicators. These can be abstracted into 10 representative feature variables in the causal graph. Then, we used the point-biserial correlation coefficient to select the 24 feature variables with the strongest correlation to hypertension. After conducting data analysis, we discovered that there are 5 feature variables that overlap between the causal feature variables and the correlated feature variables. These variables are as follows and 4 of them are shown in Figure 5.


$$SDFeature\;52\;\left(SD\;of\;TD\left(sdPPG_{c}-dPPG_{\text{valley}}\right)\right)$$

$$QDFeature\;52\;\left(QD\;of\;TD\left(sdPPG_{c}-dPPG_{\text{valley}}\right)\right)$$

$$RFeature\;52\;\left(Range\;of\;TD\left(sdPPG_{c}-dPPG_{\text{valley}}\right)\right)$$

$$MEFeature\;52\;\left(Mean\;of\;TD\left(sdPPG_{c}-dPPG_{\text{valley}}\right)\right)$$

$$MEFeature\;47\;\left(Mean\;of\;TD\left(sdPPG_{c}-PPG_{\text{peak}}\right)\right)$$

**Figure 6.**
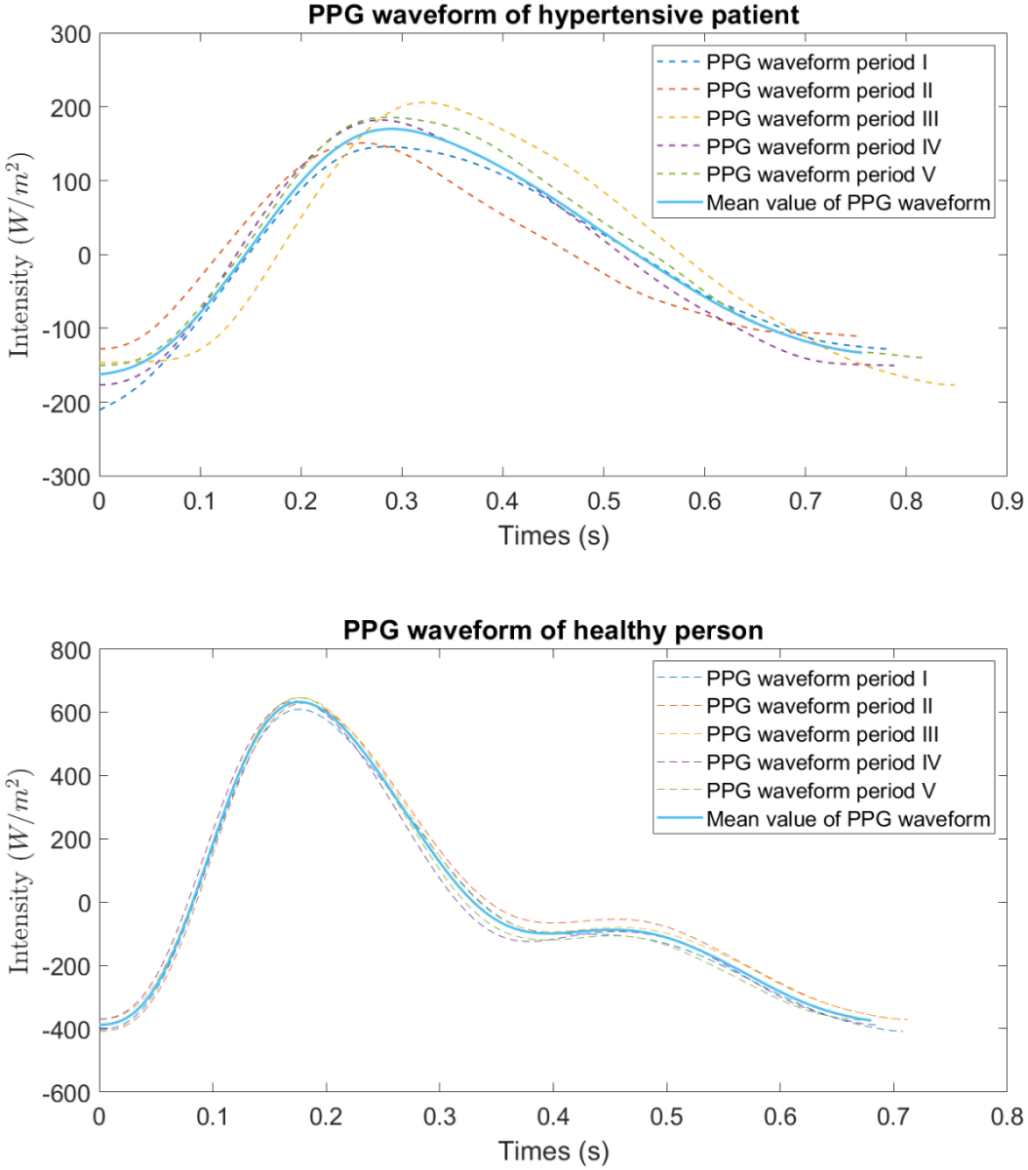
Comparison of PPG waveforms between healthy people and hypertensive patients. PPG: photoplethysmogram.

**Figure 7.**
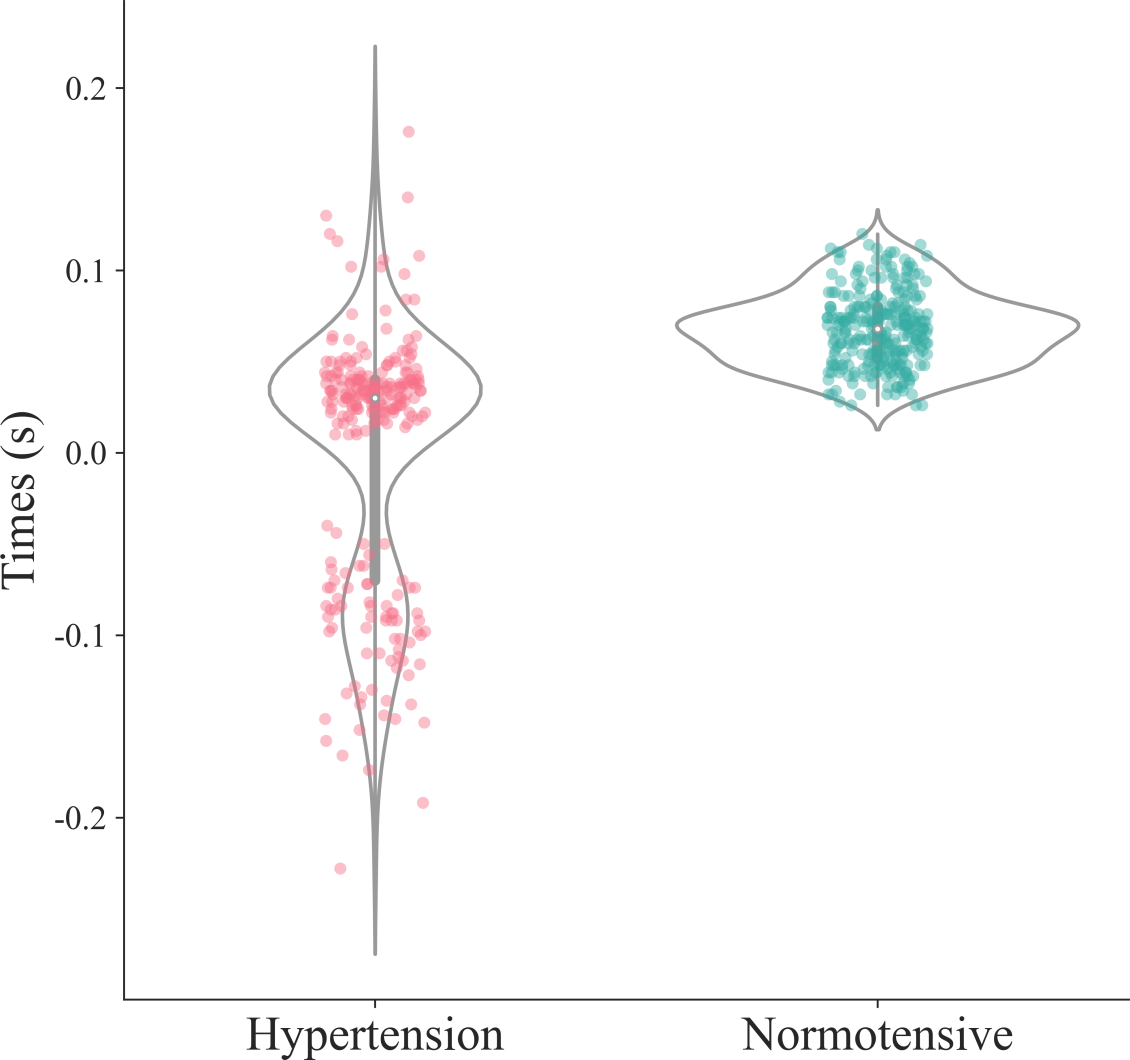
Scatter distribution of feature 52 for normotensive subjects (green) and hypertensive subjects (red).

Furthermore, we selected the representative samples from the groups of hypertensive patients and healthy people for comparative analysis. The PPG waveform analysis diagrams of hypertensive patients and healthy people are shown in Figure 6, and the scatter plots of feature 52 are shown in Figure 7. Then, based on the analysis of feature 52’s position in PPG signals, we observed that in hypertensive patients, the peak of the c-point on sdPPG may occur earlier compared to healthy individuals. This could be a possible reason as to why feature 52 is strongly correlated with hypertension and is considered to have a strong causal relationship with hypertension.

Finally, it is important to note that further research and validation are necessary to confirm the relationship between feature 52, the c-point on sdPPG, and hypertension. These findings may provide valuable insights into potential markers for hypertension and contribute to the understanding of its pathophysiology.

### Causal Graph

**Figure 8.**
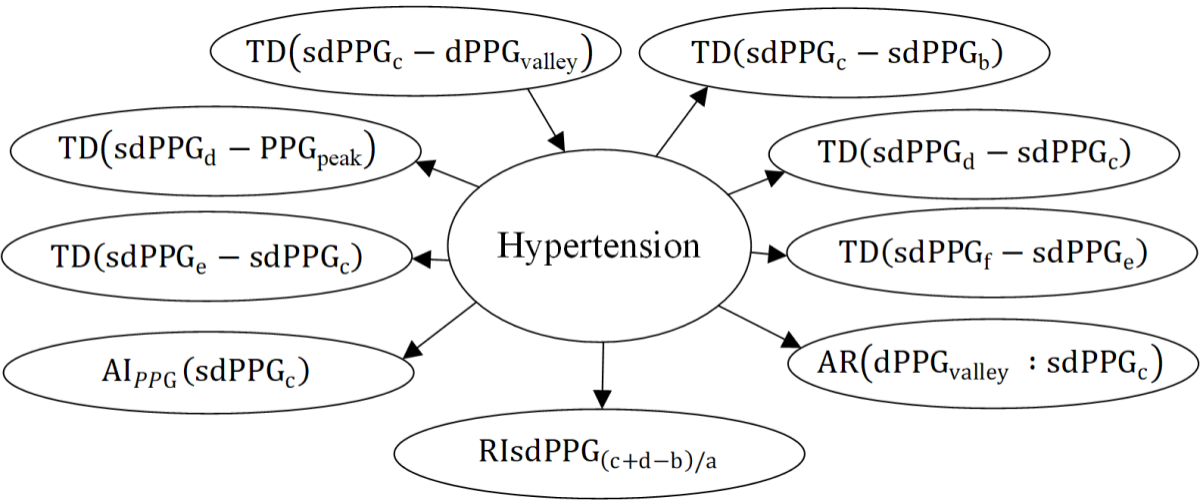
Causal subgraph of hypertension and the features calculated with their standard deviation. AI: absolute intensity ; AR: area under the PPG curve; dPPG: the first derivative of PPG; P-R: precision-recall; PPG: photoplethysmogram; RI: physiological meaningful relative index; sdPPG: the second derivative of PPG; TD: time duration.

In this study, considering the potential disturbance to the causal graph caused by randomly partitioning the data into training and testing sets, we used the idea of 10-fold cross-validation and causal strategy I to mitigate such interference. After applying the aforementioned procedures, we obtained a total of 6 causal subgraphs under different metrics. In addition, due to space constraints, this paper only presents the causal subgraphs under the standard deviation and range indicators, as shown in Figures8  9, respectively. It is observed that the feature variables directly causally associated with the risk of hypertension vary across different indicators. Based on the principle of majority rule, we applied causal strategy II to obtain the final causal graph, as depicted in Figure 10 .

**Figure 9.**
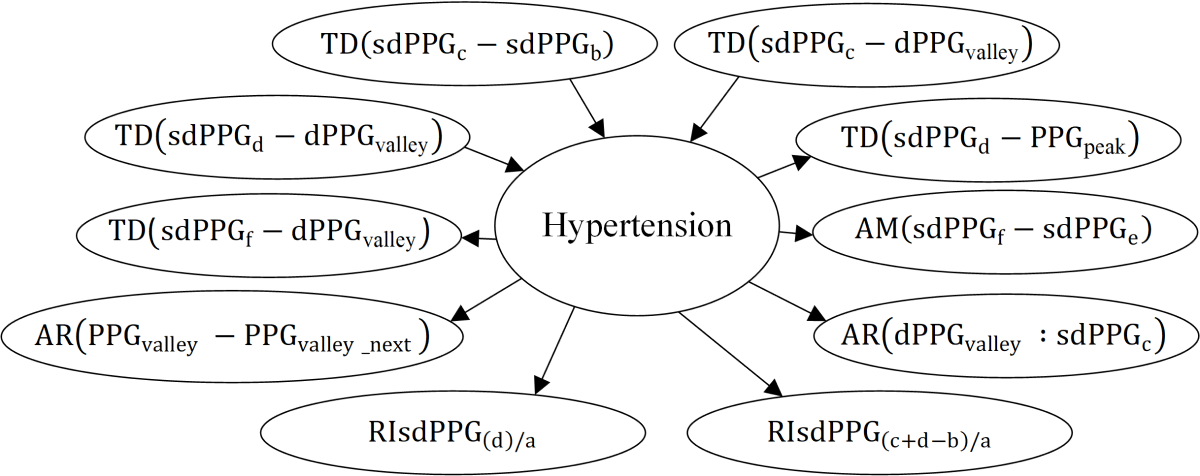
Causal subgraph of hypertension and the features calculated with their range. AM: amplitude; AR: area under the PPG curve; dPPG: the first derivative of PPG; P-R: precision-recall; PPG: photoplethysmogram; RI: physiological meaningful relative index; sdPPG: the second derivative of PPG; TD: time duration.

**Figure 10.**
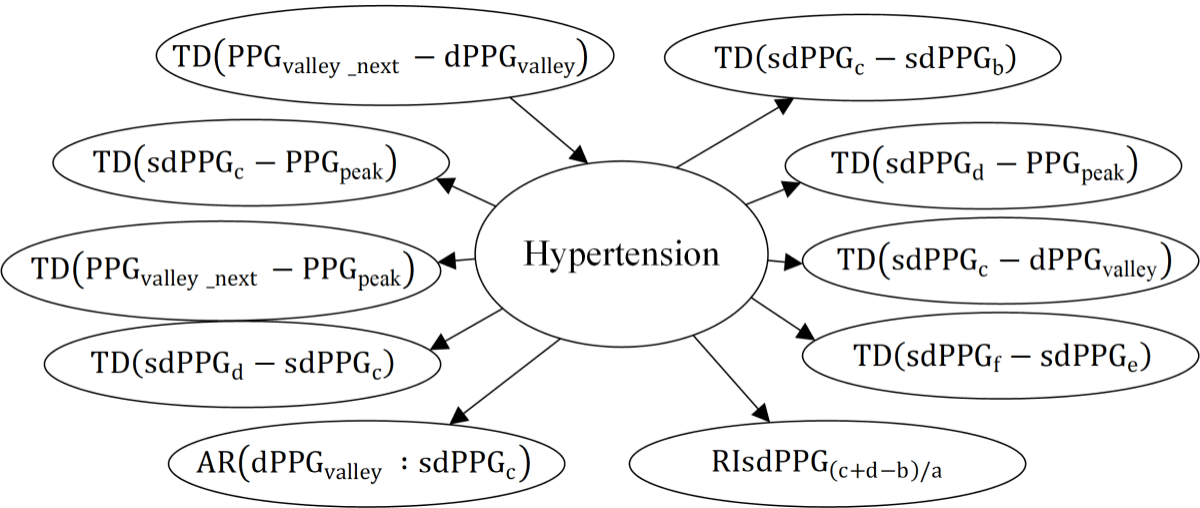
Final causal graph. AR: area under the PPG curve; dPPG: the first derivative of PPG; P-R: precision-recall; PPG: photoplethysmogram; RI: physiological meaningful relative index; sdPPG: the second derivative of PPG; TD: time duration.

### Hypertension Classification Results

In this subsection, we used multiple classifier algorithms for hypertension classification prediction. First, we primarily utilized logistic regression and other classification algorithms based on causal feature variables for hypertension classification. The classification performance is presented in Table 2. We found that the logistic regression algorithm exhibited the best predictive performance with an accuracy of 0.89, precision of 0.92, recall of 0.82, and *F*_1_-score of 0.87. Both the accuracy and accuracy rate are relatively high, which means that our classification prediction model can accurately predict hypertensive patients and healthy people , and the probability of making errors in the judgment of hypertensive patients is low ; the *F*_1_ -score further proves the above conclusion. In addition, a higher recall rate indicates that most patients with high blood pressure can be correctly predicted.

**Table 2.** Causality-based classification performanc e.

Algorithm	Accuracy	Precision	Recall	*F*_1_-score
Random forest	0.86	0.90	0.77	0.83
Decision tree	0.78	0.76	0.78	0.76
Naive Bayes	0.80	0.95	0.58	0.72
Logistic regression	0 .89	0 .92	0 .82	0 .87

**Figure 11.**
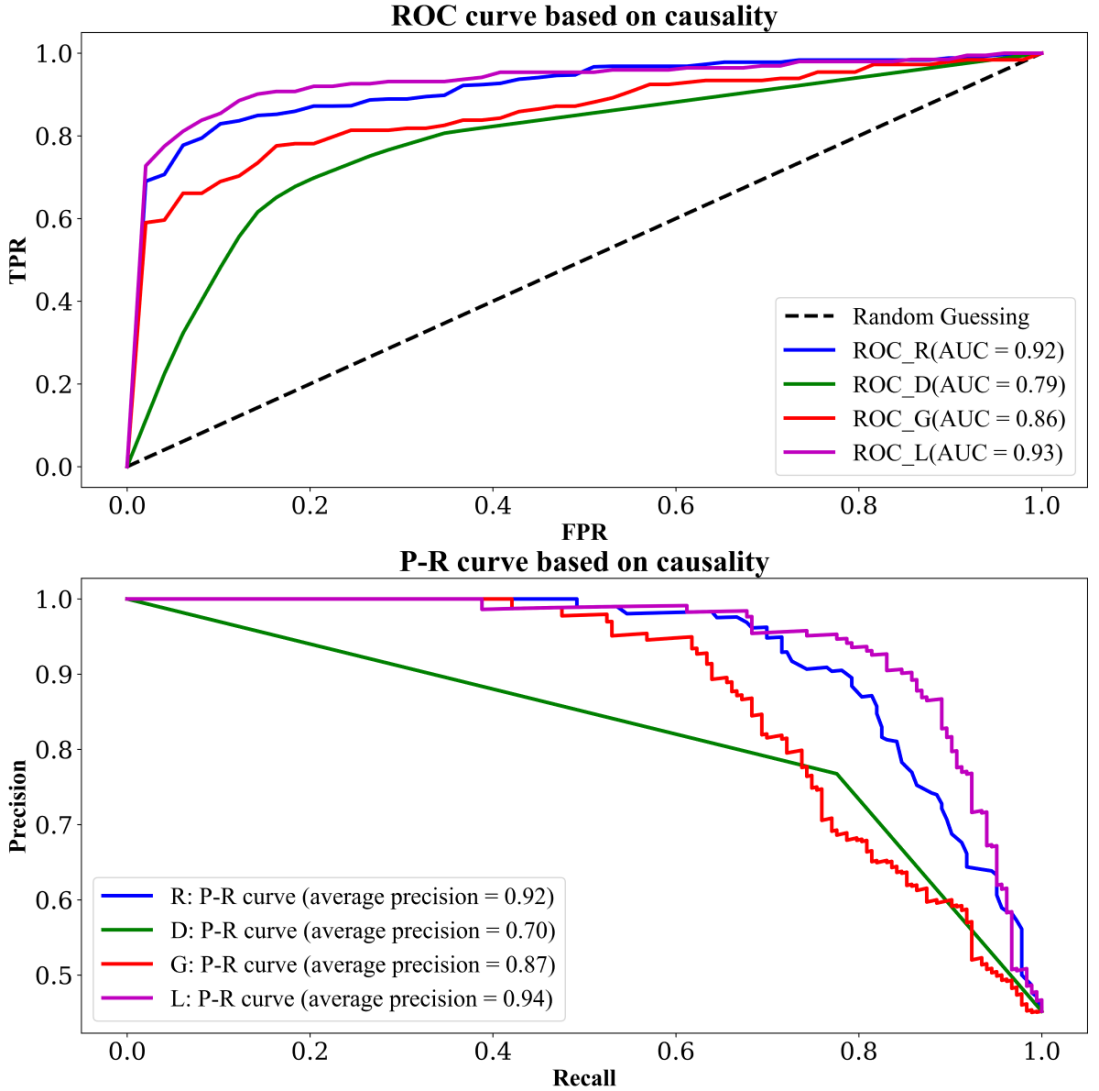
The ROC curve (top panel) and P-R curve (bottom panel) of hypertension detection based on causal features with different machine learning algorithms: the blue curve represents random forest (R), the green curve represents decision tree (D), the red curve represents naive Bayes (G), and the purple curve represents logistic regression (L). AUC : area under the receiver operating characteristic curve ; FPR: false positive rate ; P-R: precision-recall; ROC: receiver operating characteristic; TPR: true positive rate.

Subsequently, Figure 11 illustrates the receiver operating characteristic curve and precision- recall curve of the classifier algorithms. The purple line represents the logistic regression classification algorithm. It can be observed that the area under the curve of this logistic regression classification algorithm is higher than that of other classification algorithms in both receiver operating characteristic and precision-recall curves.

**Figure 12.**
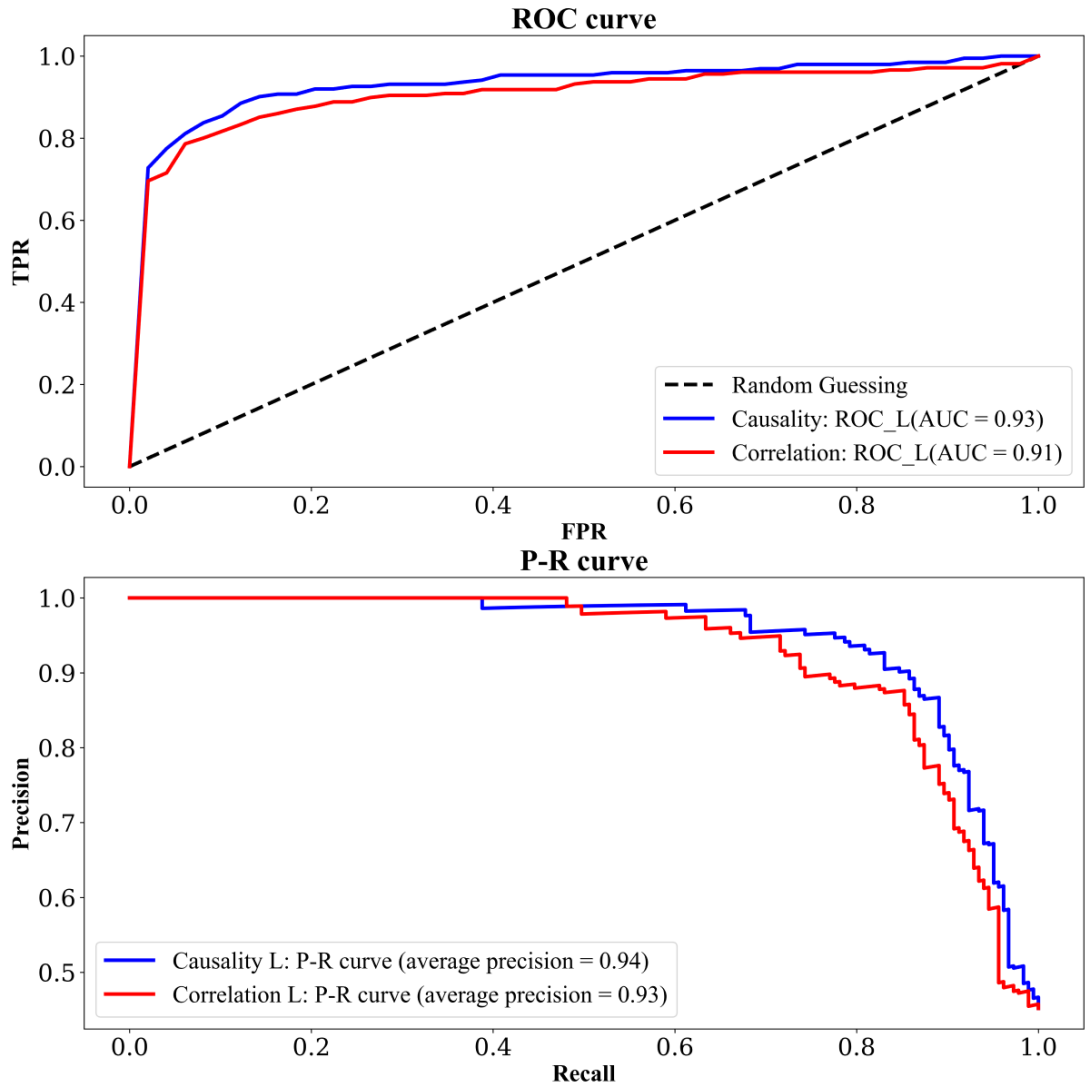
The ROC curve (top panel) and P-R curve (bottom panel) for the best classifier of causality and correlation: the blue curve represents the logistic regression classifier based on causality, while the red curve represents the logistic regression classifier based on correlation. AUC : area under the receiver operating characteristic curve; FPR: false positive rate; P-R: precision-recall; ROC: receiver operating characteristic; TPR: true positive rate.

Finally, we compared the classification performance based on causal feature variables with that based on correlated feature variables, as shown in Table 3. We found that the best performance in terms of the 4 evaluation metrics was consistently achieved by the classification algorithm based on causal feature variables. This finding is also consistent with the results presented in Figures12  13 . The se findings imply that the causal characteristics we screened have certain mining value in the field of hypertension prediction.

**Table 3.** Classifier performance compariso n.

Algorithm		Accuracy	Precision	Recall	*F*_1_ -score
**Causality**					
	Random forest	0.86	0.90	0.77	0.82
	Decision tree	0.78	0.76	0.78	0.79
	Naive Bayes	0.80	0.95	0.58	0.72
	Logistic regression	0.89	0.92	0.82	0.87
**Correlation**					
	Random forest	0.79	0.81	0.72	0.75
	Decision tree	0.72	0.68	0.72	0.69
	Naive Bayes	0.80	0.82	0.74	0.77
	Logistic regression	0.85	0.88	0.77	0.82

**Figure 13.**
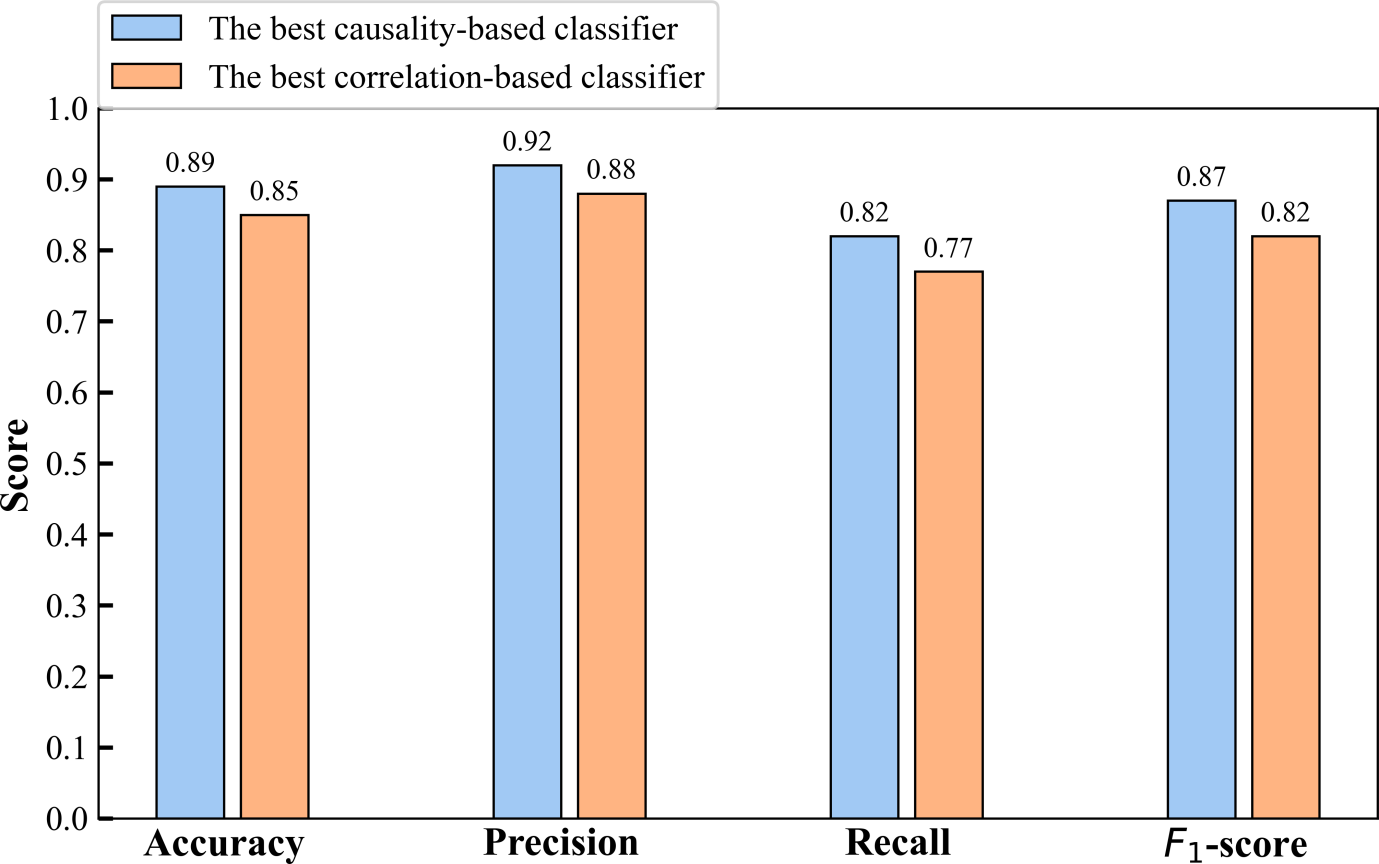
Histogram of evaluation metrics for the best classifier of causality and correlation.

## Discussion

### Principal Findings and Advantages

This study primarily explore d the relationship between feature variables extracted from ECG and PPG signals and hypertension from a causal perspective, using causal inference methods to construct causal graphs. Simultaneously, to preserve the temporal information of time series signals to the maximum extent, causal graphs were constructed separately for 6 metrics, including standard deviation, mean, range, coefficient of variation, median, and quartiles. These causal graphs were derived based on specific causal strategies, ensuring a certain degree of reliability and accuracy in the resulting causal graphs. By assessing the performance of feature variables based on causality in hypertension risk classification prediction against those based on correlation, we validated the reliability of causality-based feature variables compared to correlation-based ones.

Specifically, when selecting feature variables strongly associated with hypertension, both causal inference and correlation coefficient –based methods performed similarly. However, when the association between feature variables and hypertension was weak, causal inference methods tended to select more reliable feature variables compared to correlation-based methods. This is the reason why feature variables based on causality outperformed those based on correlation in hypertension risk prediction. Additionally, we found that feature 52’s derived variables exhibited significant differences in distribution between the hypertensive and healthy subject groups under multiple metrics. This may provide potential value and insights for subsequent pathological mechanism analysis.

### Comparison to Prior Work

T his study conducted exploratory analysis, initially focusing on the correlation analysis between hypertension and blood pressure based on the medical information mart for intensive care ( MIMIC) database. Typically, the gold standard for diagnosing hypertension is SBP and DBP, where subjects are considered hypertensive when SBP exceeds 140 mmHg or DBP exceeds 90 mmHg. Nevertheless, when clustering analysis was performed on 24-hour dynamic blood pressure data collected from patients, we observed that the blood pressure distribution of hypertensive and nonhypertensive subjects did not exhibit significant differentiation or stratification; instead, they appeared mixed. After analysis, we attributed this phenomenon to factors such as patients taking antihypertensive medications, being in specific states, or incorrect device wear, which indirectly reflects the limitations of blood pressure measurement. Second, we previously conducted causal analysis [16] using data collected from a self-generated database of 30 individuals. Causal analysis was primarily carried out under the mean metric, resulting in limited preservation of temporal information. However, it still revealed significant differences in the distribution of feature 52 between the hypertensive and healthy subject groups, consistent with the findings of this paper.

### Limitations and Future Work

There were some limitations to this study. First, our work primarily focuse d on binary classification to distinguish hypertensive patients from healthy individuals. However, hypertension can be categorized into different stages, such as stage 1, stage 2, and stage 3, based on blood pressure level and disease condition. Secon d, the population used could have been more diverse in terms of race and ethnicity. In our future work, we will consider conducting clustering of the features to distinguish different stages of hypertension, and we will validate the work on larger and more diverse subject populations to be able to draw more general conclusions.

### Conclusion

In this study, we explored the feasibility of predicting the risk of hypertension using causal inference methods. First, we constructed causal graphs using the GES algorithm and 10-fold cross-validation approach under each indicator. We then applied corresponding causal strategies to obtain the optimal causal graphs for each indicator. Finally, we merged the causal graphs from different indicators into a final causal graph based on the majority rule. After selecting the feature variables, we used classifiers including random forests, decision trees, naive Bayes, and logistic regression to predict hypertension. Overall, combining various indicators, we found that most classifiers based on causal features have better classification performance than classifiers based on correlation features. To the best of our knowledge, this study represents the first attempt to introduce causal inference methods in hypertension prediction, providing a new perspective for understanding the physiological mechanisms of hypertension.

## Abbreviations

AI: absolute intensity
AM: amplitude
AR: area under the PPG curve
AUC: area under the receiver operating characteristic curve
BP: blood pressure
DAG: direct acyclic graph
DBP: diastolic blood pressure
dPPG: the first derivative of PPG
ECG: electrocardiogram
GES: greedy equivalence search
P-R: precision-recall
PPG: photoplethysmogram
PTT: pulse transit time
RI: physiological meaningful relative index
RRI: R-R interval
SBP: systolic blood pressure
sdPPG: the second derivative of PPG
TD: time duration
